# Rhabdomyolysis due to Multiple Wasp Stings

**DOI:** 10.1155/2012/486724

**Published:** 2012-09-16

**Authors:** K. Ito, S. Imafuku, J. Nakayama

**Affiliations:** ^1^Department of Dermatology, Almeida Hospital, 1509-2 Miyazaki, 2-Chome, Oaza, Oita 870-1195, Japan; ^2^Department of Dermatology, Fukuoka University Hospital, 6-45 Momochihama, 3-Chome, Sawara-ku, Fukuoka, Japan

## Abstract

Wasp sting is a relatively common arthropod assault, but is sometimes fatal because of anaphylaxis. Rhabdomyolysis is a serious condition, with destruction of striated muscles, and can be induced by various causes such as drugs, heart attacks, CRASH syndrome, and viper bites. Mass envenomation by multiple wasp stings can also cause rhabdomyolysis followed by acute renal failure, although it is extremely rare. We herein report a case who had an anaphylaxis-like reaction and rhabdomyolysis due to multiple wasp stings.

## 1. Introduction

Rhabdomyolysis due to wasp stings is quite a rare condition and is caused by mass envenomation by multiple wasps. Although anaphylaxis appears rapidly, since rhabdomyolysis has a slow onset, cautions are required and a repeated laboratory evaluation and checking of muscle pain are needed in case of multiple wasp stings. Early diagnosis and optimal hydration were effective for rhabdomyolysis.

## 2. Case Presentation

**Figure 1:**
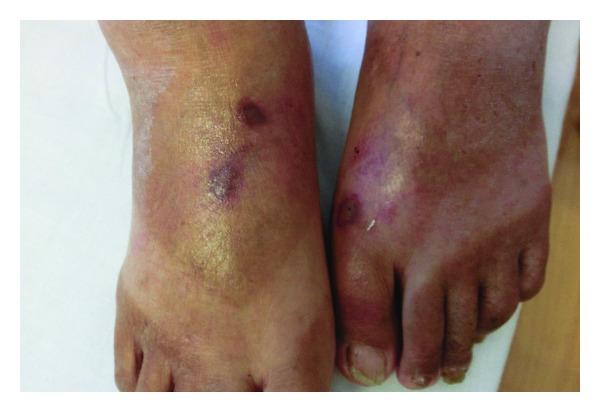
Clinical appearance of the stung sites at hospitalization. Central white necrosis and surrounding erythema were found on dorsum of feet. The patient was stung in fifteen places.

A 75-year-old Japanese man was stung by wasps on the head and the extremities while cutting down a tree in the countryside. He was referred to our clinic because of a headache and severe pain at the sites of stings. A total of 15 sting marks with central necrosis and surrounding erythema were found on his head and extremities (Figure 1). He was conscious (Glasgow Coma Score of 14), but had low blood pressure (BP-60/42 mmHg) with coldness of his extremities. He had not had a history of wasp stings. Laboratory investigations revealed leukocytosis (white cell count 14,900 *μ*L^−1^) and renal and hepatic dysfunction: blood urea nitrogen (BUN) 19 mg/dL, serum creatinine (Cr) 1.46 mg/dL, aspartate aminotransferase (AST) 483 IU/L, alanine aminotransferase (ALT) 136 IU/L, lactate dehydrogenase (LDH) 452 IU/L, and creatinine kinase (CK) 120 IU/l. Rapid transfusion of Ringer's lactate solution and intramuscular epinephrine (0.3 mg) resolved the shock, and the circulation became stable. He was also given hydrocortisone (250 mg) intravenously for prevention of late-onset anaphylaxis. On the next day, he complained of severe spontaneous muscle pains in all extremities, where he got the multiple stings. Laboratory findings were consistent with rhabdomyolysis: BUN 28 mg/dL, Cr 1.39 mg/dL, AST 696 IU/L, ALT 441 IU/L, LDH 940 IU/L, CK 10790 IU/L, serum aldolase 69.7 IU/L (normal 1.7–5.7 IU/L), serum myoglobin 5790 ng/mL (normal <60 ng/mL), and urine myoglobin 130000 ng/mL (normal <10 ng/mL). 30 hours after being stung, the man's CK peaked to a level of 17360 IU/L. He was hydrated with Ringer's lactate solution to keep urine output more than 1 mL/kg/hour. His pain, serum myogenic enzymes, and renal function recovered within 5 days, and he was discharged on the seventh hospital day. After a month, all his laboratory findings were normalized, but his skin lesions remained, with blackish necrosis.

## 3. Discussion

Rhabdomyolysis due to wasp stings is a very rare condition, caused by the toxic effect of wasp venom without an allergic reaction [1]. Similar conditions have been reported by other insects bites worldwide, such as those by the honey bee, Africanized bee, and Hymenoptera [2–4]. Mejía-Vélez [2] reported 43 cases of acute renal failure due to multiple stings of the Africanized bee. They also showed that acute renal failure occurs as a result of rhabdomyolysis. A single sting can cause IgE-mediated anaphylaxis; however, mass stings can cause systemic reactions of toxin mediated cellular damage. The wasp venom contains active amines (serotonin and histamine), wasp kinins and histamine-releasing peptides (mastoparans) [5]. They are the cause of toxic systemic reactions, including hemolysis, coagulopathy, rhabdomyolysis, and acute renal failure in severe cytotoxicity [6]. In our case, blackish necroses were formed on the site of the sting after 1 month, possibly because of severe cytotoxicity of the wasp venom. Sixteen cases of rhabdomyolysis associated with wasp stings were reported in Japan, and fifteen cases were reported to have had skin necrosis [7, 8]. Youichi et al. reported that the skin necrosis can be a poor prognostic sign of toxic systemic reactions after wasp stings [8]. Moreover, our case also had an anaphylaxis-like reaction. It has been shown that the chemical mediator of massive wasp venom leads to anaphylaxis-like reactions [9]. Our case had an anaphylaxis-like reaction which seemed to be caused by a toxic reaction in the absence of IgE-mediated allergic reaction, because his wasp-specific IgE was within normal range and he clinically lacked wheal and respiratory symptoms.

Early treatment is essential in case of multiple wasp stings, with recognition of complicating toxin-related injury.

Treatment with epinephrine and steroids should be advised in case of anaphylaxis, but in case of toxin-related multisystem injury, optimal hydration is also indispensable to avoid renal damage caused by rhabdomyolysis in addition to those reagents [10]. Hemolysis and rhabdomyolysis are the two major factors that cause acute renal failure; therefore, they should be examinated when one sees multiple wasp stings [11]. Since acute renal failure influences the prognosis of wasp stings, rapid transfusion and dialytic support are required [12]. Successful treatment with plasma exchange has also been reported in severe cases [13]. We should consider not only anaphylaxis but also the possibility of severe toxic systemic reactions in multiple wasp stings.
